# Mammalian metabolic rates in the hottest fish on earth

**DOI:** 10.1038/srep26990

**Published:** 2016-06-03

**Authors:** Chris M. Wood, Kevin V. Brix, Gudrun De Boeck, Harold L. Bergman, Adalto Bianchini, Lucas F. Bianchini, John N. Maina, Ora E. Johannsson, Geraldine D. Kavembe, Michael B. Papah, Kisipan M. Letura, Rodi O. Ojoo

**Affiliations:** 1 Department of Veterinary Anatomy and Physiology, University of Nairobi, Nairobi, Kenya; 2 Department of Biology, McMaster University, Hamilton, ON, Canada L8S 4K1; 3 Department of Zoology, University of British Columbia, Vancouver, B.C., Canada V6T 1Z4; 4 Rosenstiel School of Marine and Atmospheric Sciences, University of Miami, Florida 33149, USA; 5 EcoTox, 2263 SW 37th Ave., #816, Miami, Florida 33145, USA; 6 SPHERE, Department of Biology, University of Antwerp, B-2020 Antwerp, Belgium; 7 Department of Zoology and Physiology, University of Wyoming, Laramie, Wyoming 82071, USA; 8 Instituto de Ciências Biológicas, Universidade Federal do Rio Grande, 96203-900, Rio Grande, RS, Brazil; 9 Department of Zoology, University of Johannesburg, Johannesburg 2006, South Africa; 10 School of Dryland Agriculture Science and Technology, South Eastern Kenya University, 90200, Kitui, Kenya; 11 Department of Animal and Food Sciences, University of Delaware, Newark, Delaware 19716, USA; 12 Kisipan M. Letura, Department of Veterinary Anatomy and Physiology, Egerton University, 20115, Njoro, Kenya

## Abstract

The Magadi tilapia, *Alcolapia grahami*, a small cichlid fish of Lake Magadi, Kenya lives in one of the most challenging aquatic environments on earth, characterized by very high alkalinity, unusual water chemistry, and extreme O_2_, ROS, and temperature regimes. In contrast to most fishes which live at temperatures substantially lower than the 36–40 °C of mammals and birds, an isolated population (South West Hot Springs, SWHS) of Magadi tilapia thrives in fast-flowing hotsprings with daytime highs of 43 °C and night-time lows of 32 °C. Another population (Fish Springs Lagoon, FSL) lives in a lagoon with fairly stable daily temperatures (33–36 °C). The upper critical temperatures (Ct_max_) of both populations are very high; moreover the SWHS tilapia exhibit the highest Ct_max_ (45.6 °C) ever recorded for a fish. Routine rates of O_2_ consumption (MO_2_) measured on site, together with MO_2_ and swimming performance at 25, 32, and 39 °C in the laboratory, showed that the SWHS tilapia exhibited the greatest metabolic performance ever recorded in a fish. These rates were in the basal range of a small mammal of comparable size, and were all far higher than in the FSL fish. The SWHS tilapia represents a bellwether organism for global warming.

The Magadi tilapia, *Alcolapia grahami*, thrives in one of the most extreme environments on earth, characterized by high pH (up to 10.0), extreme alkalinity (>300 mmol L^−1^), high temperature (>40 °C), high levels of reactive O_2_ species (>8 μmol L^−1^), unusual water chemistry with salinity close to 60% seawater, and large daily fluctuations in O_2_ levels (severe hypoxia to hyperoxia)^1^,^2^,^3^,^4^. Two isolated populations were first described by Coe^1^, one living in fast-flowing hot springs (South West Hot Springs, SWHS), the other in a cooler man-made lagoon (Fish Springs Lagoon, FSL) where the water is virtually static, contained by a retaining wall built by a local industry. Physical barriers between the sites are severe^1^, but the amount of gene flow between the two populations remains controversial^5^,^6^,^7^.

Virtually all previous physiological research has been performed on the FSL fish due to their ease of capture and proximity to the town of Magadi. It is clear that they have very unusual physiology^2^,^3^,^4^,8–12. Perhaps the most notable physiological adaptation is 100% ureotelism; this is the only known teleost fish which excretes entirely urea and no ammonia^8^. Furthermore, routine metabolic rate is high^10^,^12^,^13^, and about 50% of it appears to be spent on acid-base regulation^11^. However, the two populations exhibit clear morphological differences, with the SWHS fish appearing more streamlined and having a much higher mass-specific gill area than the FSL fish^14^. Little else is known about the SWHS fish, apart from the facts that they are also ureotelic^5^ and have very high mitochondrial respiration rates^15^.

In the present study, we further characterized the two habitats, and compared the temperature tolerance and metabolic performance of the two populations. Based on the very high daily temperature and more energetically-challenging environment of the SWHS fish, and the limited physiological and anatomical knowledge summarized above, we hypothesized that both temperature tolerance and metabolic performance of the former would be exceptional. Indeed the current data show that both upper critical temperature and aerobic metabolic capacity of the SWHS tilapia surpass those of any other teleost fish.

## Results and Discussion

### Habitat Characteristics

**Figure 1:**
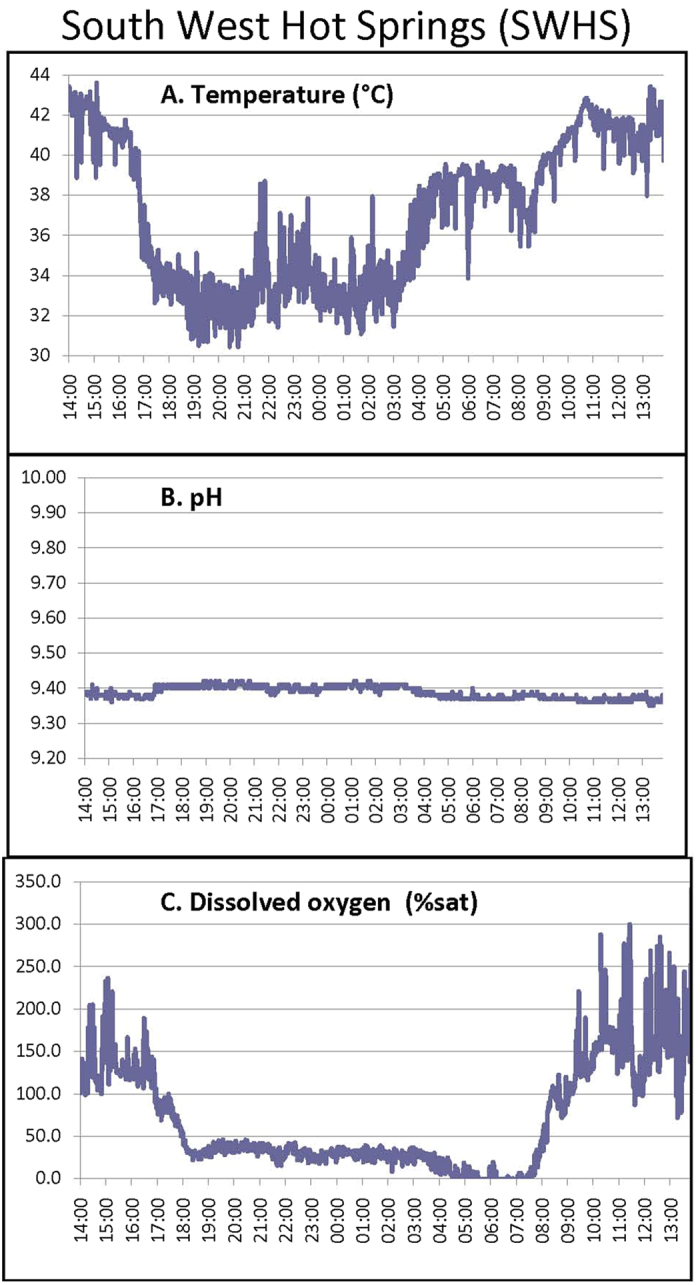
Continuous 24-h record of (A) water temperature, (B) pH, and (C) dissolved O_2_ (% saturation) at the SWHS site on Aug. 5, 2013.

**Figure 2:**
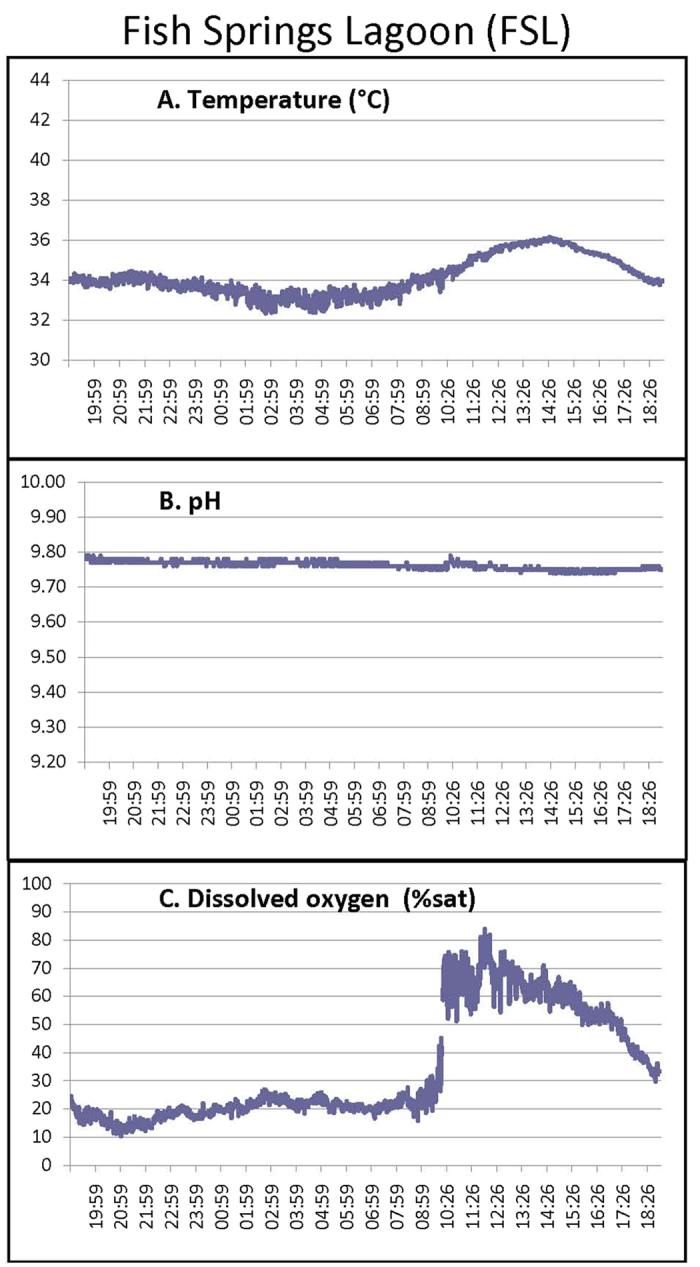
Continuous 24-h record of (A) water temperature, (B) pH, and (C) dissolved O_2_ (% saturation) at the FSL site on Aug. 1, 2013.

We characterized the diurnal temperature, dissolved O_2_, and pH profiles at the two sites (FSL, Aug. 1; SWHS, Aug. 5, 2013) in the coolest month of the year, taking care to place the probe in locations where the fish were present at mid-day. At the SWHS site where the very shallow (<12 cm), fast-flowing water is under direct sunlight, temperature varied dramatically over the day from 43 °C at 14:00 to 32 °C during the night (Fig. 1). Additionally, we routinely observed fish moving through thermal spring plumes at 43.7–44.2 °C, though not staying there. Fish were never observed in a 44.8 °C plume. Dissolved O_2_ varied from super-saturation during daylight hours to hypoxia during the night and anoxia at dawn, whereas water pH was stable at ~9.40. At FSL, where the current is negligible and the water is deeper (~1 m) but again under direct sunlight, conditions were much more constant with diurnal temperature variations of 33–36 °C, dissolved O_2_ variations of 30–80% saturation, and stable pH ~ 9.75 (Fig. 2). Other aspects of water chemistry were also very different at the two sites, with much higher ions, osmolality, total CO_2_, and titratable alkalinity at SWHS (Table 1), yet lower pH, in accord with a previous report^5^. Far more avian predators were also seen at SWHS.

### Critical Temperatures

Field Ct_max_, measured at lakeside using a standardized protocol^16^ in SWHS fish freshly caught from water at 40–41 °C, was 45.6 ± 0.1 °C (N = 8), the highest ever recorded for a fish (Table 2). FSL fish were also very temperature tolerant: the comparable field Ct_max_ for FSL fish caught from 33 °C water was 43.6 ± 0.1 °C (N = 8). In both cases, field Ct_min_ values were approximately 30 °C lower than Ct_max_. After transportation to the laboratory and holding at 33 °C in their respective waters for 4 days, Ct_max_ had dropped by 1.9 °C, and Ct_min_ by 3.2 °C in SWHS fish, whereas these values were essentially unchanged in FSL fish.

### Routine Metabolism

**Figure 3:**
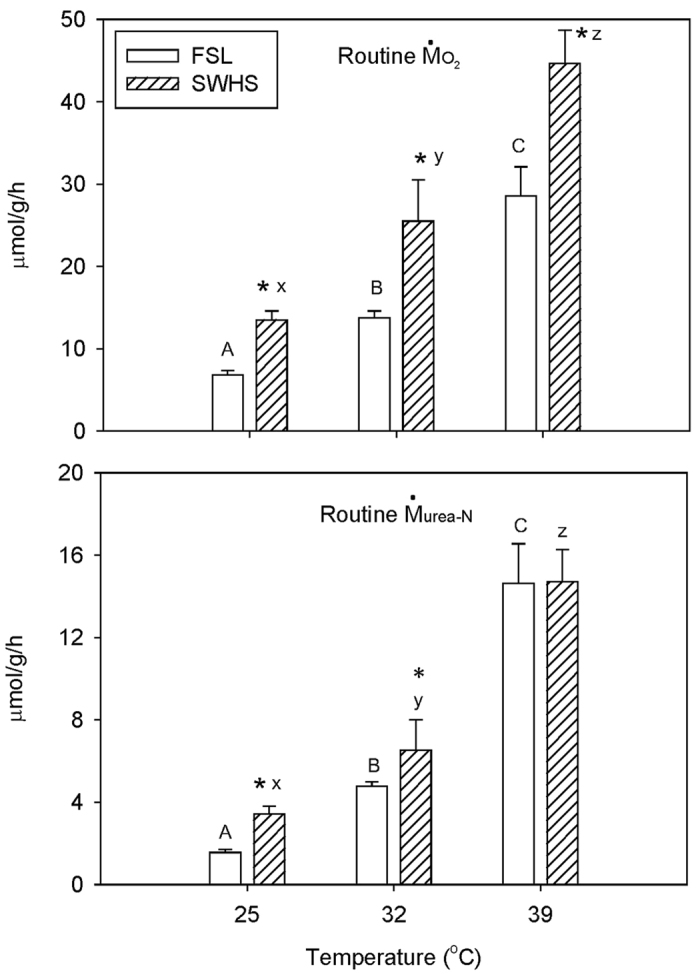
The influence of temperature on (A) routine MO_2_ and (B) routine M_Urea-N_ in the laboratory in Magadi tilapia from SWHS and FSL. Means ± 1 SEM (N = 5–7). For (**A**) routine MO_2_ the overall effects of both population and temperature (2-way ANOVA) are significant (P < 0.05); interaction effects are not significant. For (**B**) routine M_Urea-N_, the overall effects of both population and temperature (2-way ANOVA) are significant (P < 0.05); interaction effects are not quite significant (P = 0.052). Rates for FSL fish sharing the same letters are not significantly different (P > 0.05). Rates for SWHS fish sharing the same letters are not significantly different (P > 0.05). Asterisks indicate significant differences (P < 0.05) between FSL and SWHS fish at the same temperature.

Routine MO_2_, measured in the laboratory at 25 °C, 32 °C, and 39 °C using Tusker chamber respirometers^10^, was significantly higher by 1.4–2.3 fold in the SWHS fish at all three temperatures (25 °C, 32 °C, and 39 °C) (Fig. 3A). Routine M_Urea-N_, measured simultaneously, was higher only at 25 °C and 32 °C (Fig. 3B). These laboratory MO_2_ and M_Urea-N_ values for FSL tilapia were in the same general range as for previous reports on “resting” FSL fish at comparable temperatures^10^,^11^,^12^. The much higher MO_2_ values for SWHS fish were approximately double those predicted by meta-relationships with temperature for fish in general^17^. Routine metabolism increased to a greater extent with temperature (2 comparisons across 3 temperatures) in FSL fish [MO_2_ Q10 = 2.78 (2), M_Urea-N_ Q10 = 4.94 (2)] than in SWHS fish [MO_2_ Q10 = 2.46 (2), M_Urea-N_ Q10 = 2.97 (2)].

Routine MO_2_ was also measured in the field on freshly caught fish using Tusker chambers immersed in the lake, to hold ambient temperature. Routine field MO_2_ in SWHS fish was 87.7 ± 12.0 (15) μmol g^−1^ h^−1^ at a water temperature of 41 °C. These routine rates for SWHS fish in the field are truly exceptional; a similarly sized small mammal, the 6-g adult pygmy mouse (*Baiomys taylori*), has an identical routine metabolic rate (87.1 ± 9.8 μmol g^−1^ h^−1^) at an air temperature of 30–33 °C (thermoneutral zone)^18^. Routine MO_2_ in FSL fish in the field was 4-fold lower, 22.2 ± 1.5 (10) μmol g^−1^ h^−1^ at 33 °C. Routine M_Urea-N_ in the field scaled in proportion: 21.5 ± 3.3 (15) μmol g^−1^ h^−1^ at 41 °C in SWHS fish versus 5.8 ± 0.6 (10) μmol g^−1^ h^−1^ at 33 °C in FSL fish.

### Swimming Performance and Metabolism

**Figure 4:**
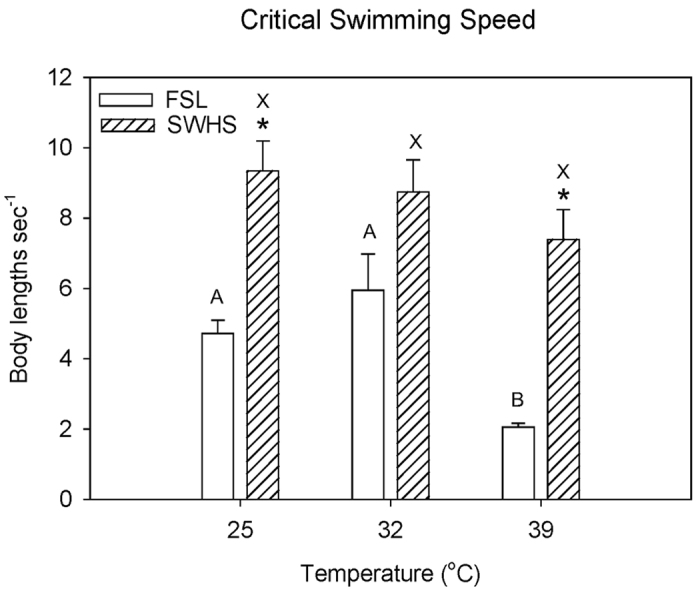
The influence of temperature on critical swimming speed (U_crit_) in Magadi tilapia from SWHS and FSL. Means ± 1 SEM (N = 5–7). The overall effects of both population and temperature (2-way ANOVA) are significant (P < 0.05); interaction effects are not significant. Values for FSL fish sharing the same letters are not significantly different (P > 0.05). Values for SWHS fish sharing the same letters are not significantly different (P > 0.05). Asterisks indicate significant differences (P < 0.05) between FSL and SWHS fish at the same water temperature.

**Figure 5:**
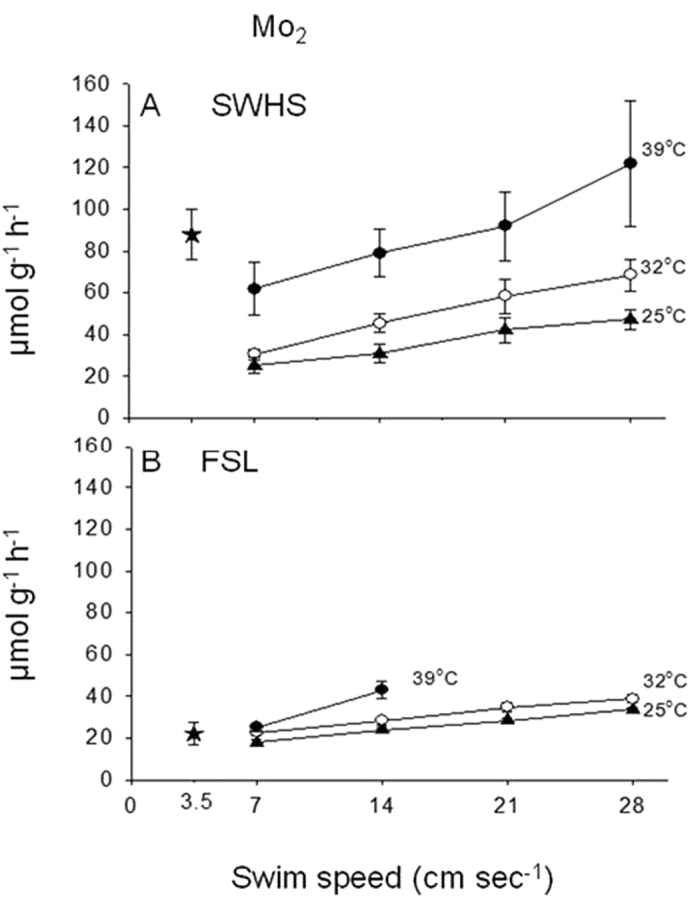
The influence of temperature on MO_2_ during swimming at increasing speeds in Magadi tilapia from (A) SWHS and (B) FSL. Means ± 1 SEM (N = 5–7). The overall effects of population, temperature, and swimming speed (3-way ANOVA) are all significant (P < 0.05); interaction effects are not significant. Also shown (as stars) are the routine MO_2_ values measured in the field for freshly caught fish (SWHS, N = 15, at 41 °C; FSL, N = 10, at 33 °C).

Critical swimming speed^19^ (U_crit_, 7.5–9.5 body lengths sec^−1^) in Blazka-style swim tunnels^20^ was high and independent of temperature (25, 32, 39 °C) in the SWHS fish, but significantly lower at both 25 °C and 39 °C in FSL fish, collapsing to only about 2 body lengths sec^−1^ at 39 °C (Fig. 4). In both populations MO_2_ increased approximately linearly with swimming speed, but the rates associated with any swimming speed at any temperature were higher (by 1.1–2.5-fold) in the SWHS fish (Fig. 5). In contrast to MO_2_, M_Urea-N_ did not increase with swimming speed in either population, though again rates were generally higher in SWHS fish (Supplementary Fig. S1). The lack of increase in M_Urea-N_ with increased aerobic metabolic rate during swimming was seen previously in FSL tilapia after exhaustive exercise^9^; possible explanations include either a switch away from amino-acid based fuels and/or inhibition of urea synthesis to reduce costs during exercise.

Anecdotally, we noticed in both populations, that if an air bubble was provided in the swimming respirometer after exhaustion, the fish would continuously breathe air for up to 40 min, presumably to help satisfy the demands of excess post-exercise O_2_ consumption.

Q10 values for MO_2_ during swimming, calculated for individual speeds, averaged 2.00 ± 0.26 (8 comparisons across 3 temperatures) in SWHS fish indicating a continuing ability to increase metabolism with temperature in the face of exercise, whereas in FSL fish the mean Q10 value during swimming was only 1.35 ± 0.07 (6 comparisons across 3 temperatures), indicating limited capacity. Mean Q10 values for M_Urea-N_ during swimming were 2.15 ± 0.53 (8) for SWHS and only 1.30 ± 0.21 (6) for FSL, indicating similar limitation. Notably these Q10 patterns contrasted with those for routine metabolism reported above which increased more quickly with temperature in FSL fish than in SWHS fish.

**Figure 6:**
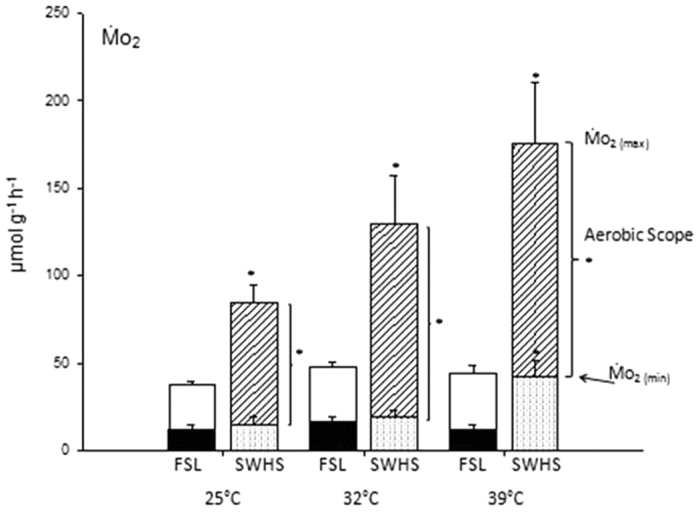
The effect of temperature on calculated MO_2(min),_ MO_2(max),_ and aerobic scope in Magadi tilapia from SWHS and FSL. Means ± 1 SEM (N = 5–7). The overall effects of population (2-way ANOVA) are significant (P < 0.05) for all three parameters, whereas those of temperature are significant only for MO_2(max),_ but there are significant interaction effects for MO_2(min)_ and aerobic scope. Asterisks indicate significant differences (P < 0.05) between FSL and SWHS fish at the same temperature.

Values for MO_2(min)_ (calculated rate of O_2_ consumption at zero activity), MO_2(max)_ (calculated rate of O_2_ consumption at maximum sustainable swimming activity) and aerobic scope (the difference between these two values) (Fig. 6) illustrate the dramatic differences between the two populations, and the exceptional capacity of the SWHS fish. MO_2(min)_, MO_2(max)_, and aerobic scope values for FSL tilapia did not differ across temperatures (25, 32, 39 °C); factorial aerobic scope was approximately 3. The relatively high measured routine MO_2_ at 39 °C (Fig. 3B) consumed much of the aerobic scope of the FSL fish, explaining their poor swimming performance at this temperature. In contrast MO_2(max)_, and aerobic scope steadily increased with temperature in SWHS fish (Fig. 6), while routine MO_2_ (Fig. 3B) remained close to MO_2(min)_, accounting for only a small fraction of the aerobic scope at all temperatures. Interestingly, MO_2(min)_ only differed from the FSL values at 39 °C, but MO_2(max)_, and aerobic scope were significantly greater at all three temperatures. Factorial aerobic scope in SWHS tilapia was about 6 at 25 °C and 32 °C, falling to 4 at 39 °C.

MO_2(max)_ at 39 °C in SWHS tilapia was 175.4 ± 34.6 (6) μmol g^−1^ h^−1^. It is impressive that this fish can sustain an MO_2(max)_ equal to about one-third of the MO_2(max)_ (507 μmol g^−1^ h^−1^) of the pygmy mouse^18^,^21^. We believe that these rates for SWHS tilapia are indicative of the greatest metabolic performance ever recorded in a fish of comparable size. The obvious comparison is with tunas, but there appear to be no published data for 3-g tunas, and they are not endothermic at this size. It is also doubtful whether even adult tuna ever reach body temperatures of 39 °C. The smallest tuna studied appears to be a 24-g kawaka (*Euthynnus affinis*) which exhibited an MO_2(max)_ of about 94 μmol g^−1^ h^−1^ at a Ucrit of about 4 body lengths sec^−1^ at 24° 22; application of a standard teleost allometric scaling coefficient^17^ would raise this to 145 μmol g^−1^ h^−1^ for a theoretical 3-g tuna. The previous record for highest MO_2(max)_ in a teleost appears to be 164 μmol g^−1^ h^−1^ in 0.019-g larvae of the damselfish *Chromis atripectoralis* swimming at a Ucrit >30 body lengths sec^−1^ at 30 °C^23^; this would scale to only 57 μmol g^−1^ h^−1^ for a theoretical 3-g larva.

### Perspectives

At least in part, this exceptional metabolic performance is explained by the very high area, thin diffusion distance, and high morphometric diffusing capacity for O_2_ (D_O2_) in the gills of *Alcolapia grahami*; D_O2_ is twice as high in the SWHS fish as in the FSL fish, and is second only to that in tuna on a mass-specific basis^14^. Mitochondrial respiration rates are also considerably higher than in other fish, but only to the extent expected because of the higher temperature^15^. Other key parts of the O_2_ delivery system (cardiac output, blood O_2_ characteristics, tissue vascularization) have yet to be examined. The metabolic differences observed could be phenotypic, genotypic or both. At present, it is unclear whether the FSL and SWHS populations are genetically distinct^5^,^6^,^7^. With respect to the current debate about the O_2_-and capacity-limited thermal tolerance (OCLTT) hypothesis^24^,^25^, the pattern of aerobic metabolism in FSL fish (Fig. 6) does not fit the idealized OCLTT model. Nevertheless, encroachment of routine MO_2_ on aerobic scope coupled with declining performance at the highest temperature (39 °C) is in accord with the theory. The pattern for SWHS fish may or may not fit the model because aerobic scope continued to increase to 39 °C (Fig. 6); experiments closer to lethal temperatures (Table 2) will be required to see if aerobic scope eventually declines as Ct_max_ is approached. The SWHS tilapia lives in a fluctuating thermal environment which comes within 3 °C of this Ct_max_ on a daily basis (Fig. 1, Table 2), and is clearly at the mercy of ambient air temperatures. Already classified by IUCN^26^ as “threatened (vulnerable)”, it will be a bellwether organism for studying the impacts of climate change. All these are exciting areas for future investigation on this unique teleost athlete.

## Methods and Materials

### Fish Capture and Habitat Characterization

Research was performed under a research and ethics clearance permit (NCST/RR1/12/1/MAS/99) from the National Council for Science and Technology (NCST Kenya), and with the permission of the Magadi Soda Foundation. Experiments were carried out in accordance with the approved guidelines of NCST Kenya and experimental protocols were approved by the Animal Use and Ethics Committee of the Faculty of Veterinary Medicine, University of Nairobi. All foreign researchers were licensed by NCST and formally appointed as visiting researchers at the University of Nairobi. Collections were made under permission from the Dept. of Fisheries, Ministry of Fisheries (Kenya). All surviving fish were returned to their original collection sites.

Adult Magadi tilapia, *Alcolapia grahami* (2.97 ± 0.14 g, 6.41 ± 0.12 cm fork length) were collected by beach seine from two sites at the edge of Lake Magadi, Kenya, in July and August, 2013: Fish Spring Lagoons (FSL) (GPS coordinates = 1°53′30.2″S, 36°18′09.9″E) and South West Hot Springs (SWHS) (2°00′04.0″S, 6°13′55.2″E). Water chemistry at the two sites (Table 1) was determined by previously described methods^5^. The daily cycle of water temperature, pH, and dissolved O_2_ was continuously recorded for 24 h at the two sites (FSL, Aug. 1; SWHS, Aug. 5, 2013) using a multi-parameter meter and probe system (HI 9828, Hanna Instruments, Woonsocket, RI, USA). Fish were studied either shortly after capture at lakeside or after transport by vehicle (FSL = 15 min; SWHS = 90 min), together with water from the two sites, to a field laboratory set up in a classroom of Magadi Secondary School.

### Measurements of Critical Temperatures

Upper (Ct_max_) and lower (Ct_min_) critical temperatures were determined by a standardized protocol^16^ in which temperature was either increased or decreased at a linear rate of 0.3 °C min^−1^ and loss of equilibrium was the endpoint. Temperature was monitored continuously by a Symphony SP70P probe and meter (VWR, Radner, PA, USA) referenced to a precision thermometer serial 210620, traceable to NIST standards (H-B Instrument, Trappe, PA, USA). Ct measurements were made either on freshly collected fish or on fish which had been held for 4 days at 33 °C in their source water in the laboratory, and were designated as “field” or “lab” respectively. During the 4-day holding, the fish were fed to satiation daily with US Sera Goldy Colour Spirulina (Sera North America Inc., Montgomeryville, PA, USA). Water was changed daily.

### Respirometry

After capture, fish for respirometry experiments were held overnight without feeding in their appropriate water at 33 °C in the laboratory. Gut clearance occurred during this time. Experiments commenced the next morning, and were always performed using water from the appropriate source. In these trials, O_2_ was measured using a portable WTW Oxi325 Oximeter (Weilheim, Germany); urea-N determinations and calculations of O_2_ consumption (MO_2_) and urea-nitrogen excretion (M_Urea-N_) rates were performed as described previously^10^,^11^. This species is capable of supplementary air-breathing via a physostomous swimbladder^4^,^12^,^13^, but access to air was not provided in any of the respirometry experiments. Temperature coefficients (Q10 values)^17^ for MO_2_ and M_Urea-N_ were calculated based on group means. Fish weights and body lengths (nose to fork of caudal fin) were measured after completion of respirometry experiments.

Routine rates of MO_2_ and M_Urea-N_ were measured in amber 500-ml bottles (“Tusker chambers”)^10^ fitted with aeration devices. In the laboratory, fish were allowed to settle for 1 h in the chambers at the chosen test temperature (25, 32, or 39 °C), then a water sample was taken for urea-N analysis. A final sample was taken after 0.3–3 h, depending on temperature. The respirometer was sealed in the middle of the flux period for a sufficient time to allow depletion of O_2_ to no less than 60% saturation, and then aeration was resumed. Blanks without fish were run simultaneously. In the field, a similar procedure was used to measure routine rates, but freshly collected fish were employed, and the respirometers were incubated directly in the lake so as to maintain ambient temperature, which was 33 °C in FSL and 41 °C in SWHS at the time of these measurements.

Swimming respirometry was performed using the 3.2-L Blazka-style swim tunnels described by Wilson *et al*.^20^ which were calibrated using an Onicon flowmeter, model F-1100 (Clearwater, Florida, USA). The four respirometers were submerged, in pairs, in two temperature-controlled baths (200-L) filled with the appropriate water. In each run, three were used for fish swimming, and the fourth as a blank at the same velocities. Each was fitted with an aeration device. Fish were allowed to settle for 1 h at about 3 cm sec^−1^, and then the respirometer was flushed (0.25 h), and the first test velocity was set to 7 cm sec^−1^ for a 1-h swimming period. The respirometer was then flushed again (0.25 h), during which time the velocity was increased to 14 cm sec^−1^, with subsequent parallel 1-h swimming periods at 21 and 28 cm sec^−1^, each followed by 0.25-h flushes. Thereafter, further progressive speed increments of 7 cm sec^−1^ were applied for 0.5-h swimming periods until the fish exhausted. These 7 cm sec^−1^ increments represented about 1.1 body lengths sec^−1^ (typical fish length = 6.0–6.7 cm). Exhaustion was defined as failure of the fish to leave the rear screen and start swimming again, after the velocity had been briefly stopped and restarted three times. The exact time of the failure was recorded and used in the calculation of critical swimming speed (U_crit_) by the method of Brett^19^. Only the first four 1-h periods were used for respirometry, as this duration was necessary for accurate determination of M_Urea-N_. In the middle of each period, aeration was suspended and the respirometer was sealed for MO_2_ determination, using a duration in which O_2_ depletion occurred to no less than 60% saturation. The subsequent periods were used only to assess swimming performance. This protocol was adopted to fit into a 9-h period (daylight hours only) in light of security concerns (bandits) when travelling to and from our laboratory.

### Data Analysis

For each fish, MO_2_ was plotted against swimming speed. As with other high performance fish swimming at high temperature^22^, the relationships were better described by linear rather than exponential relationships for most fish (33 out of 37), so linear regression was used throughout to predict MO_2(min)_ at 0 body lengths sec^−1^ and MO_2(max)_ at U_crit_, with the difference representing aerobic scope. Data have been expressed as means ± 1 SEM (N). Data were transformed as required to achieve normal distribution and homogeneity of variance prior to analysis by three-way ANOVA (population x temperature x swimming speed) or two-way ANOVA (population x temperature) as appropriate, followed by the Holm-Sidak post hoc test (P < 0.05) to identify individual differences.

## Additional Information

**How to cite this article**: Wood, C. M. *et al*. Mammalian metabolic rates in the hottest fish on earth. *Sci. Rep.* **6**, 26990; doi: 10.1038/srep26990 (2016).

## Supplementary Material

Supplementary Information

## Figures and Tables

**Table 1:** Measured water chemistry at Fish Springs Lagoon (FSL) and South West Hot Springs (SWHS) in August 2013.

	FSL	SWHS
pH	~9.75	~9.40
Titratable Alkinity (to pH 4.0, mmol L^−1^)	230	378
Total CO_2_ (mmol L^−1^)	165	282
Na^+^ (mmol L^−1^)	392	674
Cl^−^ (mmol L^−1^)	125	190
K^+^ (mmol L^−1^)	2.7	4.3
Ca^2+^ (mmol L^−1^)	0.10	0.05
Mg^2+^ (mmol L^−1^)	0.002	0.001
Osmolality (mosm kg^−1^)	513	880

**Table 2:** Maximum (Ct_max_ ) and minimum (Ct_min_) critical temperatures for loss of equilibrium in Magadi tilapia collected from South West Hot Springs (SWHS) or Fish Springs Lagoon (FSL).

	Ct_max_ (°C)	Ct_min_ (°C)
SWHS Magadi tilapia field^a^	45.6 ± 0.1	17.6 ± 0.4
SWHS Magadi tilapia lab^b^	43.7 ± 0.4	14.4 ± 0.1
FSL Magadi tilapia field^a^	43.6 ± 0.5	13.2 ± 0.3
FSL Magadi tilapia lab^b^	44.5 ± 0.0	12.4 ± 0.3
Sheepshead minnow lab^c^	45.1 ± 0.1	
Sheepshead minnow lab^d^	44.2 ± 0.1	11.3 ± 0.2
Yucatan pupfish lab^e^	45.3 ± 0.1	
Common killifish lab^f^	42.5 ± 0.2	9.6 ± 0.2

Values are means ± 1SEM for all fish, with N = 8 for Magadi tilapia. Comparisons are made to other fish species which previously held the record for the world’s most high temperature tolerant fish.

^a^*Alcolapia grahami* Measurements made shortly after capture from 40–41 °C water for SWHS fish and from 33 °C water for FSL fish.

^b^*Alcolapia grahami* Measurements made after the fish were held for 4 days at 33 °C in their respective waters in the laboratory.

^c^*Cyprinodon variegatus variegatus* Measurements made in the laboratory after fish were held for 30 days with a daily 5 °C thermoperiod ranging from 37–42 °C^27^.

^d^*Cyprinodon variegatus variegatus* Measurements made in the laboratory after fish were held for 30 days at 38 °C^27^.

^e^*Cyprinodon artifrons* Measurements made in the laboratory after fish were held for 8 days with a daily 15 °C thermoperiod ranging from 26–41 °C^28^.

^f^*Fundulus heteroclitus* Measurements were made in the laboratory after fish were held for 21 days at 34 °C^29^.
