# Supplementary material for: Mammalian metabolic rates in the hottest fish on earth
Source: Sci Rep. 2016 Jun 3;6:26990. doi: 10.1038/srep26990 (PMC4891707; doi:10.1038/srep26990)
Supplement: Supplementary Information [file srep26990-s1.pdf]

Supplementary Material

Mammalian metabolic rates in the hottest fish on earth

Authors:

Chris M. Wood[1,2,3,4], Kevin V. Brix[1,2,3,5], Gudrun De Boeck[1,6], Harold L. Bergman[1,7], Adalto Bianchini[1,8], Lucas F. Bianchini[1,8], John N. Maina[1,9], Ora E. Johannsson[1,3], Geraldine D. Kavembe[1,10], Michael B. Papah[1,11], Kisipan M. Letura[1,l2], and Rodi O. Ojoo[1]

Addresses:

[1]Department of Veterinary Anatomy and Physiology, University of Nairobi, Nairobi, Kenya

[2]Department of Biology, McMaster University, Hamilton, ON, Canada L8S 4K1

[3]Department. of Zoology, University of British Columbia, Vancouver, B.C., Canada V6T 1Z4

[4]Rosenstiel School of Marine and Atmospheric Sciences, University of Miami, Florida 33149, USA

[5] EcoTox, 2263 SW 37th Ave., #816, Miami, Florida 33145, USA

[6]SPHERE, Department of Biology, University of Antwerp, B-2020 Antwerp, Belgium

[7]Department of Zoology and Physiology, University of Wyoming, Laramie, Wyoming 82071, USA

[8]Instituto de Ciências Biológicas, Universidade Federal do Rio Grande, 96203-900, Rio Grande, RS, Brazil

[9]Department of Zoology, University of Johannesburg, Johannesburg 2006

[10]School of Dryland Agriculture Science and Technology, South Eastern Ken[illegible] University, 90200, Kitui, Kenya

[11]Department of Animal and Food Sciences, University of Delaware, Newark, Delaware 19716, USA

[12]Kisipan M. Letura, Department of Veterinary Anatomy and Physiology, Egerton University, 20115, Njoro, Kenya.

*Correspondence to:

Chris M. Wood

Orcid ID: 0000-0002-9542-2219

Department of Zoology

University of British Columbia

Vancouver, B.C., Canada

Phone: 1-604-827-1576

FAX: 1- 604-822-2416

Email: woodcm@zoology.ubc.ca

Supplementary Figure Legend

Supplementary Figure S1. The influence of temperature on $M_{Urea\text{-}N}$ during swimming at increasing speeds in Magadi tilapia from (A) SWHS and (B) FSL. Means ± 1 SEM (N = 5-7). The overall effect of population (3-way ANOVA) is significant ($P < 0.05$) whereas those of temperature and swimming speed are not; interaction effects are not significant. Also shown (as stars) are the routine $M_{Urea\text{-}N}$ values measured in the field for freshly caught fish (SWHS, N = 15, at 41°C; FSL, N = 10, at 33°C).

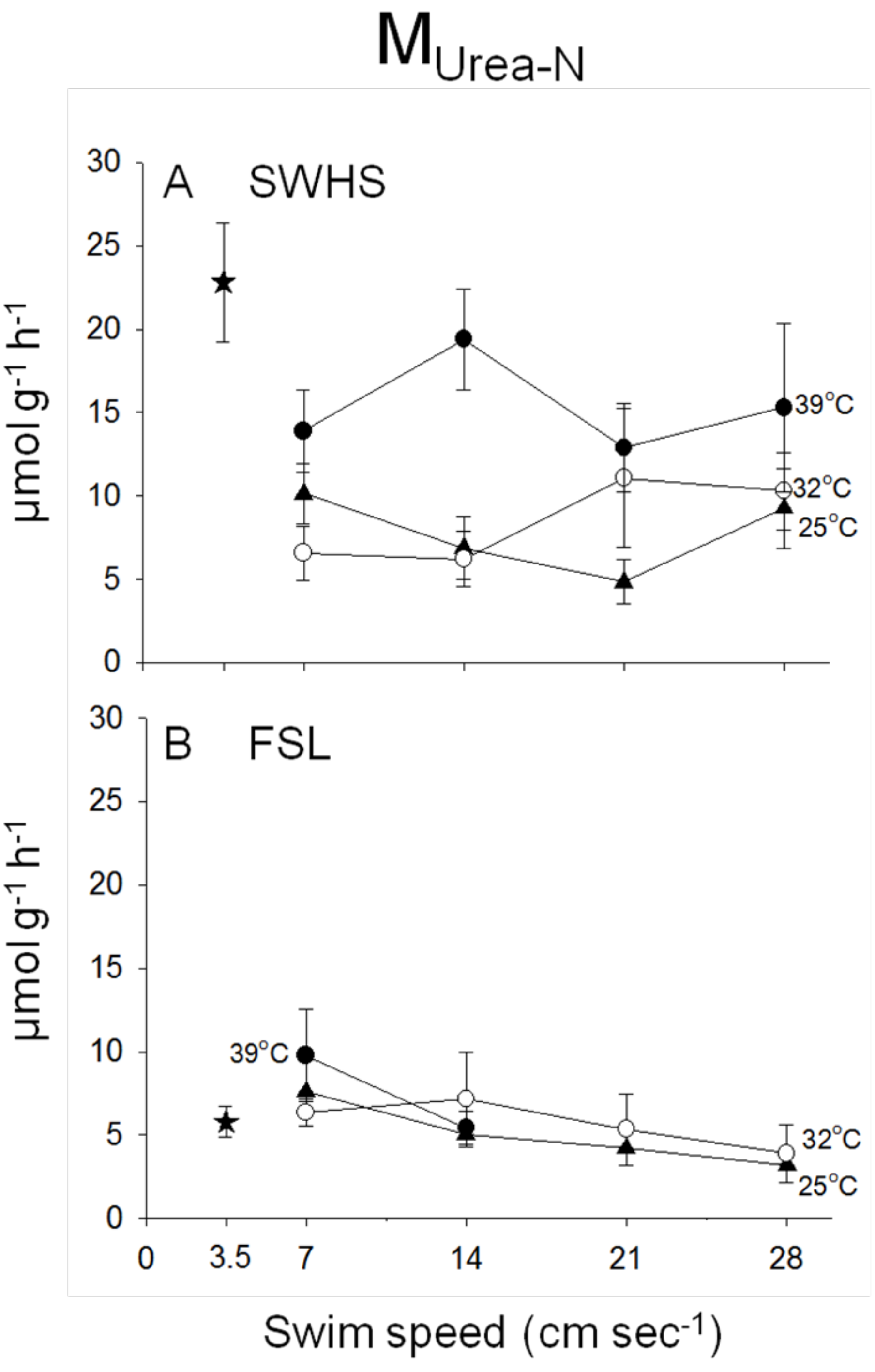

M_{Urea-N}
A SWHS
B FSL
μmol g-1 h-1
30
25
20
15
10
5
0
39°C
32°C
25°C
0
3.5
7
14
21
28
Swim speed (cm sec-1)
